# Biodegradable Polymer-Based Drug-Delivery Systems for Ocular Diseases

**DOI:** 10.3390/ijms241612976

**Published:** 2023-08-19

**Authors:** Ta-Hsin Tsung, Yu-Chien Tsai, Hsin-Pei Lee, Yi-Hao Chen, Da-Wen Lu

**Affiliations:** 1 Department of Ophthalmology, Tri-Service General Hospital, National Defense Medical Center, Taipei 11490, Taiwan; happyshino5@gmail.com (T.-H.T.); 400010419@mail.ndmctsgh.edu.tw (Y.-C.T.); doc30848@gmail.com (H.-P.L.); keanechen18@gmail.com (Y.-H.C.); 2 Department of Ophthalmology, Taoyuan Armed Forces General Hospital, Taoyuan 325, Taiwan

**Keywords:** biodegradable polymers, nanomedicine in ophthalmology, ocular drug delivery, controlled drug release, sustained drug delivery, anterior segment disorders, posterior segment disorders, ocular bioavailability, drug delivery system

## Abstract

Ocular drug delivery is a challenging field due to the unique anatomical and physiological barriers of the eye. Biodegradable polymers have emerged as promising tools for efficient and controlled drug delivery in ocular diseases. This review provides an overview of biodegradable polymer-based drug-delivery systems for ocular diseases with emphasis on the potential for biodegradable polymers to overcome the limitations of conventional methods, allowing for sustained drug release, improved bioavailability, and targeted therapy. Natural and synthetic polymers are both discussed, highlighting their biodegradability and biocompatibility. Various formulation strategies, such as nanoparticles, hydrogels, and microemulsions, among others, are investigated, detailing preparation methods, drug encapsulation, and clinical applications. The focus is on anterior and posterior segment drug delivery, covering glaucoma, corneal disorders, ocular inflammation, retinal diseases, age-related macular degeneration, and diabetic retinopathy. Safety considerations, such as biocompatibility evaluations, in vivo toxicity studies, and clinical safety, are addressed. Future perspectives encompass advancements, regulatory considerations, and clinical translation challenges. In conclusion, biodegradable polymers offer potential for efficient and targeted ocular drug delivery, improving therapeutic outcomes while reducing side effects. Further research is needed to optimize formulation strategies and address regulatory requirements for successful clinical implementation.

## 1. Introduction

The human eye is a highly intricate organ protected by robust anatomical and physiological barriers, rendering it an immune-privileged organ, and impeding systemic circulation [1]. The eye’s structure can be classified into two primary segments: the anterior segment and the posterior segment. The anterior segment encompasses the cornea, conjunctiva, aqueous humor, iris, ciliary body, and lens, while the posterior segment primarily comprises the vitreous humor, sclera, retina, choroid, and optic nerve. The intricate anatomy and physiology of the eye impose inherent and unique barriers that serve to protect against environmental toxins and microorganisms. However, these barriers also pose significant challenges in achieving effective ocular drug delivery. Traditional methods of drug administration, including topical eye drops and ointments, often exhibit an inadequate bioavailability and limited therapeutic outcomes [2]. These limitations arise from various factors such as tear turnover, tear film dynamics, and the presence of ocular barriers such as the cornea, conjunctiva, and blood–retinal barriers [3,4,5]. Consequently, frequent high-dose administrations are typically required, contributing to patient noncompliance and treatment failure.

These limitations have necessitated the demand for innovative strategies to enhance drug delivery to targeted ocular tissues and improve therapeutic efficacy. Biodegradable polymers have emerged as promising candidates for ocular drug-delivery systems (DDSs) due to their ability to undergo degradation, thereby enabling the controlled and sustained release of drugs at the targeted site. Through their controllable degradation, they enable precise and sustained drug release at the designated site. Moreover, the inherent properties of biodegradable polymers, such as their biocompatibility, adaptability, and versatility in formulation, align effectively with the unique requirements imposed by the eye’s structure and function. Utilizing biodegradable polymer-based DDSs offers the potential for multiple benefits, including enhanced drug stability, an extended residence time in ocular tissues, improved drug bioavailability, a reduced dosing frequency, and the possibility for targeted delivery to specific ocular tissues, thereby bridging the gap between the eye’s natural barriers and therapeutic needs [6].

This review delivers an in-depth examination of the utilization of biodegradable polymers in ocular DDSs, focusing primarily on a broad range of both natural and synthetic polymers, including but not limited to collagen, chitosan, gelatin, poly(lactic-co-glycolic acid), poly(lactic acid), and poly(caprolactone). We scrutinize the methods used to formulate nanoparticles, microparticles, and hydrogels, providing a detailed exploration of their mechanisms of drug encapsulation and release and their real-world applications. Additionally, we expand the scope of the review to discuss how these polymer-based delivery systems are used to manage various ocular conditions that affect both the anterior and posterior segments of the eye, including glaucoma, disorders of the cornea, ocular inflammation, age-related macular degeneration, and diabetic retinopathy. We also critically assess the safety and biocompatibility of these systems and evaluate potential adverse effects. The aim of this review is to offer a comprehensive understanding of the application of biodegradable polymers in ocular drug delivery, to engage in a discussion about their advantages and disadvantages, and to identify potential areas for future research in this ever-evolving field.

## 2. Types of Biodegradable Polymers

The application of biodegradable polymers in DDSs is an evolving and intriguing field that warrants a closer look. Biodegradable polymers are usually made up of monomers linked together by esters, amides, or ether bonds, which can be broken down by enzymatic activity or hydrolytic processes [7]. The degradation process results in smaller molecules, like carbon dioxide, water, methane, and inorganic compounds, which can be further metabolized or excreted by living organisms. They can degrade and eventually dissolve within the body, eliminating the need for removal and reducing potential complications [8]. This property, along with their potential for controlled drug release, makes them an attractive choice for DDS design. Various types of biodegradable polymers have been utilized in the field of ocular drug delivery, each with unique properties and potential applications [9].

### 2.1. Natural Biodegradable Polymers

Natural biodegradable polymers, derived from biobased sources, serve as vital constituents in the formulation of innovative ocular DDSs. This class of polymer encompasses a diversity of substances, including polysaccharides such as cellulose derivatives, chitosan, alginate, and hyaluronic acid, along with proteins like gelatin. Delving into these polymers calls for an examination of their intrinsic properties, their specific implementations in ocular drug delivery, and their potential to revolutionize therapeutic approaches for an assortment of ocular conditions. Their unique advantage lies in their inherent biocompatibility, minimal toxicity, and beneficial interaction with biological systems, which make them ideal materials for creating ocular drug-delivery mechanisms [10]. In the subsequent sections, we will delve into some key natural biodegradable polymers.

#### 2.1.1. Cellulose Derivatives

Recognized as the most abundant biodegradable polymer, cellulose is a polysaccharide primarily synthesized by plants. It is a linear biopolymer distinguished by elongated macromolecular chains of the recurring cellobiose units [11]. Cellulose exhibits a unique capability for biodegradation, proceeding via enzymatic oxidation, predominantly through the action of peroxidase enzymes secreted by fungi and bacteria [12]. The nontoxicity of cellulose underscores its utility, making it an integral component of the naturally derived polymers extensively leveraged in ocular DDSs [13]. Methylcellulose, initially introduced in the 1940s as a viscosity control agent, has since been the subject of comprehensive scholarly scrutiny. The ensuing epochs have witnessed rigorous investigations into the utility of cellulose polymers, manifested by a plethora of animal and human studies [14,15].

Despite its advantages, the intrinsic crystalline structure of cellulose endows it with an inherent insolubility and nonfusibility in organic solvents. This poses a significant challenge to its direct utilization in the biomedical and pharmaceutical realms. However, this limitation can be strategically circumvented via the synthesis of cellulose derivatives through a range of chemical modification techniques, including esterification, etherification, or oxidation [16]. Derivatives such as methylcellulose (MC), hydroxypropyl cellulose (HPC), hydroxypropyl methylcellulose (HPMC), hydroxyethyl cellulose (HEC), and carboxymethylcellulose (CMC) are frequently incorporated in ocular formulations due to their distinct beneficial properties [17]. These polymers break down through enzymatic hydrolysis, generating monosaccharides and smaller oligosaccharides. The chemical modification processes of cellulose give rise to an array of valuable characteristics, including their water solubility, adhesiveness, film-forming ability, and emulsifying properties [18,19]. These features significantly broaden the scope of cellulose applicability within essential fields. Moreover, the swelling characteristics, chemical composition, and structural form of cellulose derivatives play crucial roles in determining the mechanisms through which the drugs enclosed in these systems are released [20,21]. Specifically, these macromolecules exhibit notable mucoadhesive properties that facilitate sustained drug release at the ocular surface, thereby optimizing the drug bioavailability and reinforcing their suitability for ophthalmic applications [22].

Cellulose and its derivatives undeniably offer promising avenues in the realm of ocular drug delivery. Nevertheless, the presence of certain challenges necessitates consistent research and development efforts to fully harness their potential. One notable challenge lies in the balancing act between achieving extended drug release and maintaining an optimal therapeutic efficacy [23]. The remarkable mucoadhesive properties of cellulose derivatives facilitate sustained drug release; however, attaining a fine-tuned balance to ensure peak therapeutic levels presents a complex task. The design of these delivery systems requires intricate calibration at each step to guarantee a steady drug-release profile, thereby enhancing therapeutic benefits while minimizing adverse effects. Moreover, the biodegradability of cellulose derivatives introduces its own set of complications. The inconsistent degradation rates of these substances can complicate the prediction of their performance in vivo, potentially affecting the drug-release profile and resulting in unforeseen therapeutic outcomes [24]. Manufacturing-related challenges, such as the need for advanced techniques for cellulose-derivative production and concerns over the scalability of these processes, can inflate costs and restrict accessibility. Furthermore, despite the recognized biocompatibility and nontoxicity of cellulose and its derivatives, there exists a potential for immunogenic reactions, especially with repeated exposure or in hypersensitive individuals. This necessitates rigorous biocompatibility testing and the vigilant monitoring of patient responses during clinical application. These complex challenges therefore represent critical areas for future investigation, aimed at advancing the use of cellulose-based systems in ocular drug delivery [17].

#### 2.1.2. Chitosan

Chitosan, a positively charged polysaccharide derived from chitin, has displayed considerable potential in the field of ocular drug delivery [25]. Regarded as the second most abundant natural biopolymer, chitosan primarily originates from the exoskeletons of crustaceans, including crabs, shrimp, and crawfish, in addition to insects. Recent studies on fermentation technology propose that a fungal cultivation could serve as an alternate source of chitin, further diversifying its availability for application in ocular drug delivery [26]. Chitosan is a linear copolymer composed of N-acetyl-glucosamine and N-glucosamine units, interconnected through β-1,4 linkages [27]. The ratio of glucosamine to acetyl glucosamine, known as the degree of deacetylation, can range between 30% and 100% and is highly dependent on the particular preparation method utilized. This degree of deacetylation critically impacts the crystallinity, surface energy, and degradation rate of chitosan. Regarding its degradation, chitosan is predominantly degraded by enzymatic reactions with enzymes like lysozyme that cleave its β-1,4 linkages, thus influencing its overall performance in the context of drug delivery [28].

Due to its cationic nature, chitosan demonstrates the capacity to engage effectively with the negatively charged cornea and conjunctiva, enabling a potential interaction with the amino groups of chitosan. This interplay could potentially augment the drug’s concentration and prolong its residence time, thereby facilitating a heightened accuracy in the application of the instilled drop solution and ensuring consistent dosing outcomes [29,30]. It is worth noting that chitosan also exhibits penetration-enhancing attributes, contributing to its ability to disrupt the tight junctions in epithelial cells, an action which significantly improves its permeability across these barriers [31,32]. As an easily accessible polymer, chitosan distinguishes itself through its nontoxicity, high biocompatibility, biodegradability, and low immunogenicity, which positions it as an optimal candidate for pharmaceutical and biomedical applications [33]. Further amplifying its therapeutic potential, chitosan possesses inherent antimicrobial properties and exhibits a mucoadhesive character [34]. Additionally, its reactive amino and hydroxyl groups render it prone to chemical modifications, thereby enabling the straightforward modulation of its physicochemical properties. Derivatives of chitosan, such as N-carboxymethylchitosan and N-carboxyethylchitosan, have been synthesized and utilized for diverse applications [35,36]. The versatility of chitosan extends to its formulation potential, which includes a broad spectrum of forms like micro- and nanoparticles, films, membranes, and gels [37,38]. Collectively, these features underscore chitosan as an exceptionally suitable polymer for ocular drug-delivery applications.

While chitosan demonstrates significant potential, certain challenges are associated with its use. As a basic polymer, chitosan’s mucoadhesive properties are confined to specific pH ranges, showing a diminished efficacy at a neutral pH compared to HEC [39]. Chitosan’s insolubility in water and alkaline media is attributed to its dense, rigid crystalline structure and the existence of robust intra- and intermolecular hydrogen bonds. It only becomes soluble in acidic solutions, specifically those with a pH less than six [40]. The pH sensitivity of chitosan hampers its broad application in drug and gene delivery due to the instability of many biomolecules at a low pH [41]. Additionally, under neutral physiological conditions, chitosan presents further constraints such as a limited water solubility and insufficient swelling properties, which limits its applications [42]. Addressing these challenges, chitosan has been subjected to modifications with specific monomers that bear supplementary reactive groups. This adjustment facilitates the assurance of mucoadhesion at a pH level of seven, a critical requirement for ocular release formulations, considering that the ocular mucus exhibits a mildly basic pH, approximating 7.8 [25,43].

#### 2.1.3. Hyaluronic Acid

Hyaluronic acid (HA), a primary component of the extracellular matrix, is an anionic glycosaminoglycan constituted by D-glucuronic acid and N-acetyl glucosamine units linked by β-1,4- and β-1,3-glycosidic bonds [44]. HA possesses multiple hydroxyl and carboxylic acid groups in conjunction with a singular amide functional group, thus facilitating a broad spectrum of chemical modifications. The eminent biocompatibility of HA stems from its endogenous nature and its wide distribution within ocular tissues. This includes the cornea, aqueous humor, iris, lens, vitreous, and retinal structures [45]. Concurrently, HA is implicated in the recuperation of the cornea, the regulation of intraocular pressure, and the migration of inflammatory cells [46,47]. Additionally, given its distinctive structure and polyelectrolyte attributes, HA exhibits particular rheological characteristics. Solutions of HA behave as non-Newtonian fluids, demonstrating phenomena such as shear thinning and viscoelasticity [48]. The viscoelastic properties of HA solutions are influenced by various factors including the molecular weight, concentration, pH, and the presence of additional molecular agents [49]. An increase in the molecular weight and concentration elevates the viscosity of the solutions, whereas the introduction of small molecular reagents like phospholipids, guanidine, and sodium chloride reduces both viscous and elastic moduli. In contrast, the addition of sugar enhances these properties. Furthermore, the viscoelasticity of HA solutions exhibits sensitivity to changes in pH as alterations in pH influence the degree of HA chain ionization and hence the intermolecular interactions among HA molecules. Modifications in ionic strength or temperature also induce a decrease in viscosity [50]. With respect to its degradation, HA undergoes enzymatic degradation primarily by three types of enzymes: hyaluronidase, β-D-glucuronidase, and β-N-acetyl-hexosaminidase. These enzymes cleave the glycosidic bonds, and this process can be influenced by factors such as pH, temperature, and enzymatic concentration and can be tailored to control the degradation rate in various biomedical applications [51].

By virtue of its acid groups, HA establishes adherence to the corneal mucin layer via noncovalent bonds, effectively emulating the adhesion behavior of the mucin glycoprotein with the sialic acid portion of eye mucin [45]. This adhesion property can be intensified by either augmenting the molecular weight or decreasing the pH of the HA solution, which subsequently prolongs the residence duration of ocular medications and boosts their utilization efficiency. Additionally, this property is bolstered by HA’s high hydration capacity, anti-inflammatory attributes, and cell permeability, albeit these features are contingent upon the pH and concentration [52]. As a long-chain hydrophilic polymer, HA exhibits an exceptional capacity to bind and retain substantial volumes of water, facilitating the formation of hydrogels. These unique characteristics of HA, encompassing bioadhesion, biocompatibility, a receptor-recognition capability, and viscoelasticity, underpin its extensive utilization across a range of ocular treatments including interventions for dry eye, the remediation of corneal wounds, the formulation of ophthalmic viscous surgical devices, and comfort agents for contact lenses [53].

Despite the considerable advantages of HA in ocular drug delivery, it is not devoid of limitations. Variability in its physicochemical properties, notably its molecular weight, can impact its biocompatibility, degradation rate, and viscoelastic properties, causing inconsistencies in the formulation of HA-based ocular DDSs [50]. Further challenges include the high viscosity of HA solutions, which complicates the formulation process and possibly impedes drug diffusion. The susceptibility of HA to enzymatic degradation by ocular hyaluronidases can also shorten its therapeutic duration [54]. While typically demonstrating an excellent biocompatibility, HA may trigger adverse reactions in some individuals, such as transient ocular discomfort, itching, and erythema. Nevertheless, the utilization of advanced formulation strategies is paving the way to overcome the inherent challenges in HA’s application for ocular drug delivery. For instance, crosslinking techniques can be employed to increase the structural integrity and stability of HA, thereby controlling its degradation rate and enhancing its functionality [55]. Another novel noncytotoxic microencapsulation platform enables the creation of HA microspheres, allowing for targeted delivery and controlled release in various applications [56]. Moreover, the integration of nanotechnology has led to the development of innovative HA-based nanocarriers, which can be tailored to optimize drug loading, release kinetics, and biocompatibility [57]. These strategic approaches are integral in unlocking the full potential of HA in the field of ocular drug delivery, opening new doors for research and therapeutic applications.

#### 2.1.4. Gelatin

Gelatin, a biodegradable and biocompatible protein obtained from collagen, is widely utilized in drug delivery due to its unique properties [58]. As a structural protein, collagen constitutes roughly 25–35% of the entire protein content in the body and is ubiquitous in the connective tissues of all vertebrates. It is found in abundance specifically within the skin, tendons, and ligaments [59]. This ubiquitously occurring protein is distinguished by its intricate hierarchical organization, characterized by a primary structure featuring a distinct, highly conserved interspecies repetition of the (Gly-X-Y)n triplet, where “Gly” represents glycine, “X” typically stands for lysine, and “Y” usually denotes hydroxyproline [60]. Each individual unit of collagen displays a secondary structure composed of three right-oriented polyproline-II helices. These units come together to create a right-oriented triple helix, indicating its tertiary structure [59]. Gelatin is derived from collagen through partial hydrolysis facilitated by acid, alkaline, or heat, thus preserving a primary structure remarkably similar to that of collagen. The degradation mechanism of gelatin primarily involves enzymatic hydrolysis, where enzymes such as collagenase and protease break down the triple helical structure into smaller peptides and amino acids.

Collagen and gelatin possess numerous advantageous characteristics, including their availability, biocompatibility, biodegradability, nontoxicity, noncarcinogenicity, reduced immunogenicity, and enhanced solubility in aqueous systems. However, gelatin is favored as a biopolymer compared to its parent protein owing to its ease of manufacture, customizability, and higher density of functional groups accessible for modifications. Gelatin-based materials do present inherent challenges, such as suboptimal mechanical properties, thermal instability, and a relatively rapid degradation time [61]. These limitations are not insurmountable and can be managed through targeted modifications. For instance, the use of crosslinkers like formaldehyde and glutaraldehyde has been shown to improve mechanical properties [62]. Specific ligands such as biotin, avidin, or peptides can be conjugated with gelatin to enhance the thermal stability and enable controlled drug release [63]. Such precise alterations significantly broaden the scope and versatility of gelatin, making it a preferred choice in the development of ocular DDSs [62].

Akin to chitosan, gelatin exhibits potent mucoadhesive characteristics, an attribute resulting from its positively charged amine groups that facilitate interactions with the negatively charged ocular mucus layer [30]. Notably, given that collagen constitutes a significant portion of the corneal stroma, the utilization of gelatin as a delivery vehicle can enhance the drug bioavailability due to its interaction with corneal and conjunctival glycoproteins. This advantage positions it favorably over invasive methods of ocular drug delivery, allowing for controlled drug delivery and a reduced dosing frequency by exploiting the properties of the gelatin matrix and the inclusion of crosslinking agents [64]. Biomaterials composed of crosslinked gelatin have earned commendation for their utilization as bioadhesives within ocular tissues, performing vital functions in the reinforcement and stabilization of retinal tissues [65]. Given its biodegradable and biocompatible nature, gelatin has also found use as a drug carrier across diverse nanoformulations. Additionally, gelatin-based biomaterials have garnered significant attention in the sphere of regenerative medicine in ophthalmology due to their unique properties [61].

#### 2.1.5. Alginate

Alginate, a naturally occurring anionic polymer, is predominantly derived from brown algae [66]. On a commercial scale, the extraction of alginates from marine brown algae involves a series of chemical treatments aimed at purging various impurities such as endotoxins, proteins, heavy metals, and other carbohydrates [67]. Alginate is a linear, unbranched, high-molecular-weight polysaccharide consisting of two uronic acids: β-D-mannuronic acid (M) and α-L-guluronic acid (G). These acids are organized into a block structure consisting of homopolymeric (MM or GG blocks) and heteropolymeric sequences (MG blocks) [68]. The polymer backbone, abundant in free carboxyl and hydroxyl groups, renders alginate an ideal substrate for chemical functionalization [69]. Alginate predominantly occurs in nature as alginic acid salts of various metal cations such as Mg^2+^, Sr^2+^, and Na^+^. The degradation of alginate is complex, involving enzymatic degradation by specific enzymes like alginate lyases, which cleave the glycosidic bonds between the uronic acid residues. Additionally, the rate at which alginate degrades exhibits a strong correlation with pH levels, experiencing accelerated degradation under conditions of highly alkaline (pH greater than 10.0) or highly acidic (pH lower than 5.0) environments [66]. Among its various forms, the sodium salt variant exhibits greater stability due to intermolecular catalysis by the C-5 carboxyl groups, thus having a longer shelf life compared to alginic acids. Therefore, sodium alginate is widely utilized owing to its superior solubility in various aqueous solvents.

The ocular environment tolerates alginate well due to its mucoadhesive behavior that extends the residence time, potentially augmenting the ocular bioavailability. The swelling of the alginate polymer chain prompts the formation of noncovalent bonds with mucin [70]. Coupled with its pH-dependent gel formation ability, alginate has been deployed as a vehicle for the controlled release of therapeutic agents. Additionally, alginate’s unique attributes such as its capability to form hydrogels in the presence of divalent cations (typically calcium), biocompatibility, nontoxicity, and capacity to sustain a moist healing environment endorse it as an ideal candidate for sustained ocular drug delivery [66]. The customizable nature of alginate hydrogels allows for the modulation of drug-release kinetics, the enhancement of drug stability, and improved patient adherence via a diminished administration frequency [71]. Alginate’s usage in ocular insert formulation also exhibits potential for localized, controlled, and sustained drug delivery through prolonged contact, improving the therapeutic effectiveness and patient comfort when compared to traditional eye drops [72]. The burgeoning domain of ocular tissue engineering and regenerative medicine also acknowledges the value of alginate. Its similarity to the natural extracellular matrix composition allows it to function as an optimal scaffold for cell proliferation and differentiation, with studies exploring its use in corneal and retinal tissue reconstruction [73,74,75].

Notwithstanding its numerous advantages, the utilization of alginate, like other biopolymers, does encompass certain drawbacks. Specifically, alginate hydrogels may display an insufficient mechanical strength and stability, constraining their applicability in some instances [76]. Moreover, as a natural substance, the properties of alginate can exhibit variability contingent upon its origin and extraction methodology. Despite these constraints, the versatility and biocompatibility of alginate underscore its potential as a promising material within the biomedical sector.

### 2.2. Synthetic Biodegradable Polymers

Synthetic polymers represent an expansive assortment of artificially created macromolecules tailored for targeted usage, especially within the biomedical domain. In contrast to their natural counterparts, synthetic polymers offer an unparalleled range of structural and functional diversity, attributed to the wide spectrum of available monomers and fabrication methodologies [77]. This adaptability enables the customization of these polymers to cater to precise biomedical requisites. The intrinsic characteristics of these synthetic polymers, encompassing their customizable structural and functional properties, biocompatibility, biodegradability, substantial drug/gene loading potential, and the ease of modifying their degradation rates, solidify their indispensability in crafting ocular DDSs [6]. A detailed examination of select synthetic biodegradable polymers such as an analysis of their intrinsic characteristics, their specific utility in ocular drug delivery, their potential to revolutionize the treatment of diverse ocular conditions, and associated challenges will be discussed in the forthcoming section, thereby facilitating a comprehensive understanding of their role within the evolving paradigm of ocular drug delivery.

#### 2.2.1. Polylactic Acid

Polylactic acid (PLA) is an aliphatic, biosourced polyester synthesized from lactic acid (2-hydroxypropionic acid) [78]. The chirality of lactic acid, which possesses two isomers—L and D—facilitates its polymerization into three unique forms: poly-L-lactic acid (PLLA), poly-D-lactic acid (PDLA), and poly-D,L-lactic acid (PDLLA) [79]. Predominantly derived from renewable resources such as starch or sugar cane via L-lactic acid-producing bacteria, the L-lactic acid forms the major portion of PLA. PLA’s crystallization can occur in three distinct forms (α, β, and γ), contingent on the composition of the optically active L- and D-enantiomers [80]. The optical purity of the lactic acid is crucial, as even minor enantiomeric impurities can significantly impact the PLA’s properties, such as their crystallinity and biodegradability. PLA is a thermoplastic material characterized by its hydrophobicity, high strength, and high modulus. Significantly, it has received approval from the Food and Drug Administration (FDA) for direct interaction with biological fluids, underscoring its ecofriendly profile. The appeal of PLA also lies in its degradability in the human body through the hydrolysis of its ester linkages into lactic acid, which subsequently undergoes metabolism in the Krebs cycle and ultimately results in the production of carbon dioxide and water, both of which can be eliminated from the body [81].

The degradation kinetics of PLA can be modulated by manipulating its molecular weight and degree of crystallinity during polymer synthesis. This adaptability augments its versatility in biomedical applications, particularly enabling its utilization for sustained drug-release paradigms [82]. Furthermore, the degradation kinetics of PLA are significantly influenced by environmental parameters, especially the pH and temperature. Notably, the degradation rate of PLA markedly decelerates under acidic conditions and exhibits an accelerating trend with an increasing temperature [83,84]. These pH- and temperature-dependent properties of PLA can be strategically exploited to tailor the half life of the PLA structure commensurate with the target tissue. Owing to its unique characteristics including its biocompatibility, biodegradability, mechanical robustness, and adaptability to various processing techniques, PLA has established itself as an essential polymer for an array of biomedical applications. It serves a pivotal role in fabricating bioresorbable sutures and scaffolds. Within the sphere of ophthalmology, PLA is utilized in a range of forms such as nanoparticles, microparticles, and implants, all of which are crucial for controlled drug release [85,86]. It also exhibits exceptional versatility in designing DDSs, having demonstrated efficacy in the delivery of both hydrophilic and hydrophobic therapeutic agents, inclusive of anti-inflammatory drugs and antibiotics, in diverse ocular applications.

While PLA exhibits numerous favorable properties, its application also brings certain challenges. The byproducts of PLA degradation can induce a localized mild inflammatory response, requiring the meticulous design of PLA-based delivery systems. Additionally, transforming PLA into specific morphologies, such as nanoparticles or films, necessitates precise process control to achieve the targeted properties. Moreover, PLA’s innate brittleness and deficient impact strength can present certain challenges. While the inclusion of nonbiodegradable additives such as nanoclays, isocyanates, peroxides, and synthetic rubbers can be employed to mitigate these issues, their use must be judiciously moderated to maintain the integrity of PLA’s biodegradable attribute [86]. Alternatively, blending PLA with other biodegradable polymers could present an effective strategy to enhance its properties or create novel PLA polymers or blends for specific applications [87,88]. Future advancements in fabrication techniques and a deeper understanding of PLA’s interaction with ocular tissues are expected to further expand its applicability in this field.

#### 2.2.2. Poly(lactic-co-glycolic acid)

Poly(lactic-co-glycolic acid) (PLGA) is a synthetic, hydrophobic copolymer resulting from the amalgamation of PLA and poly(glycolic acid) (PGA). PGA is a biodegradable semicrystalline polymer with potential for clinical application. However, its usage is restricted due to its poor solubility in conventional polymer solvents, toxic synthesis-related solvents that may interact with drugs or tissues, susceptibility to hydrolysis, and the presence of potentially harmful residual reactants. Furthermore, its inability to be shaped into films, rods, or capsules via solvent or melt techniques further constrains its application [89]. Nevertheless, the copolymerization of the PGA monomer with lactide yields the widely studied biomaterial for drug delivery, PLGA. This polymer was the first to be approved by the FDA for DDSs and remains widely used due to its notable properties [90]. PLGA is renowned for its nontoxicity, biodegradability, and biocompatibility, with the remarkable capability to be fabricated into an array of shapes and sizes while encapsulating a broad spectrum of molecule sizes [91]. In contrast to the limited solubility exhibited by pure PLA and PGA, PLGA displays enhanced solubility, being soluble in a diverse range of traditional solvents such as chlorinated solvents, tetrahydrofuran, acetone, or ethyl acetate [90].

The biodegradation process of PLGA within aqueous environments occurs via the hydrolytic cleavage of its ester linkages, leading to the generation of innocuous byproducts, namely lactic and glycolic acids, which are subsequently eliminated from the body. A distinguishing attribute of PLGA is the ability to modulate its degradation kinetics by altering the lactic acid-to-glycolic acid ratio during polymerization [92]. The inclusion of methyl side groups in PLA enhances its hydrophobicity relative to PGA, leading to a reduced hydrophilicity, lower water absorption, and slower degradation in lactide-enriched PLGA copolymers [93]. Moreover, the versatility of PLGA extends to the fine tuning of its mechanical strength, swelling characteristics, and drug-release profiles, achievable via control over the PLA-to-PGA ratio and, subsequently, the crystallinity of PLGA [93]. The introduction of crystalline PGA into the polymer matrix diminishes PLGA’s crystallinity, thereby enhancing its hydration and hydrolysis rates. Notably, a direct relationship exists between a polymer’s crystallinity and melting point and its molecular weight, underscoring the complex interplay of these factors in determining the properties of the polymer.

The inherent biocompatibility, adaptable degradation rate, protracted drug-release characteristics, and capacity to envelop a diverse range of therapeutic molecules establish PLGA as a prime candidate for the conceptualization and fabrication of ocular DDSs. However, despite being the most widely used biodegradable synthetic polymer, PLGA still has some challenges to overcome. Local inflammatory responses due to changes in ocular pH due to the production of acidic degradation byproducts require careful consideration during system design [94]. In addition, the slow degradation rate of PLGA, although beneficial for the sustained release of the drug, may result in a prolonged presence in ocular tissues, which may cause discomfort or other adverse reactions. The common issue of an initial burst release, leading to potential toxicity due to exceeding therapeutic drug concentrations, presents a challenge in balancing sustained-release profiles [95]. Ongoing research aims to further optimize PLGA-based systems for ocular drug delivery.

#### 2.2.3. Polycaprolactone

Polycaprolactone (PCL), a semicrystalline aliphatic polyester, is derived from the induced ring-opening polymerization of ε-caprolactone monomers. This hydrophobic material is marked by a slow biodegradation rate, which spans from several months to years, contingent upon factors such as the molecular weight of the polymer and the dimensions and location of the implant [96]. PCL is esteemed for its biocompatibility, low toxicity, and superior thermal stability. Despite its underwhelming mechanical attributes, its flexibility at room temperature and the ease of surface modifications allow it to be molded into various configurations. To offset its mechanical shortcomings, PCL is often modified or amalgamated with other polymers [97].

The affordability, adaptability for modification and copolymerization, and ease of processing make PCL an attractive selection for various experimental DDSs [98]. The crystalline nature of PCL bestows upon it remarkable structural integrity, even during the later stages of degradation, thereby making it a suitable material for thin-film and cellular delivery systems. Prior investigations have substantiated that PCL thin films display remarkable ocular compatibility, evoke minimal intraocular inflammation, and maintain their intricate structural attributes over prolonged periods of ocular residence [99,100]. Investigative pursuits underscore the prospective utility of PCL in ocular drug delivery. Scholarly emphasis has been directed towards the exploration of PCL-based nanoparticles for treating an array of ocular disorders, such as glaucoma [101,102,103], ocular inflammation [104,105], and keratitis [106], along with their integration into contact lenses [107], ocular implants [102,103,105,108,109], and the formation of injectable in situ hydrogels [110]. Additional research initiatives involve the evaluation of the biocompatibility of PCL nanofiber patch grafts in rabbit models [111]. Owing to the extended degradation period of PCL, a sustained drug presence is ensured, thereby positioning it as a promising material for enduring ocular DDSs.

#### 2.2.4. Polyanhydrides

Polyanhydrides (PAs), a category of synthetic biodegradable polymers, have garnered substantial interest in the realm of ocular drug delivery due to their unique biodegradation properties, exceptional biocompatibility, constant-rate drug-release kinetics, and the low toxicity profile of their degradation byproducts [112]. PAs exhibit numerous subclasses, each distinguished by the type of monomer unit linked through an anhydride bond [112,113]. Classic categories include aromatic PAs, such as poly(isophthalic anhydride) and poly(terephthalic anhydride), that contain embedded aromatic rings which confer an increased thermal, mechanical, and hydrolytic stability, consequently leading to slower degradation. Aliphatic PAs, such as poly(sebacic anhydride), are recognized by their aliphatic chains that lead to lower stability yet faster degradation. Unsaturated PAs, fabricated from aliphatic or aromatic monomers and characterized by the presence of unsaturated double or triple bonds, exhibit crystalline properties and insolubility in common organic solvents. As PA usage widens in the biomedical field, new subclasses have been created to optimize specific characteristics for individual applications. For instance, aromatic–aliphatic PAs, such as poly(carboxyphenoxy propane-sebacic acid), exemplify copolymers incorporating both aromatic and aliphatic units, striking a balance between stability and degradation rates. These polymers, being semicrystalline with less crystallinity than their aromatic counterparts but superior mechanical and thermal properties compared to aliphatic versions, provide tailored mechanical strength, degradation/erosion rates, melting temperatures, and solubility, making them well suited for a plethora of biomedical applications. On another front, crosslinked PAs feature three-dimensional polymer chain networks which augment stability and diminish degradation rates. This crosslinking methodology offers a robust avenue to modulate the physical, mechanical, and degradation properties of PAs, leading to enhanced mechanical properties, thermal stability, and resistance to solvent evaporation. Finally, fatty acid-based PAs, derived from naturally occurring fatty acids such as poly(stearic anhydride), are characterized by their superior biocompatibility and biodegradability. These various subclasses of PAs offer a wealth of materials with adjustable properties that are vital for diverse applications, particularly in DDSs [114].

PAs, owing to their high aqueous reactivity, engage in swift hydrolytic cleavage, thus generating nontoxic acidic monomeric units at controlled and predictable rates. They present a unique degradation pattern, predominantly through surface erosion, allowing a greater degree of control and predictability in drug release compared to many other biodegradable polymers. Such properties situate PAs as formidable surface-eroding carriers in ocular drug application in various forms such as biomedical implantable devices, microparticles, and nanoparticles. Their rapid hydrolytic cleavage and amenability to low-temperature processing techniques like injection molding or extrusion allow for the mass production of ocular DDSs tailored through monomer selection, composition, surface area, and additives [113]. In addition, the amenability of PAs to low-temperature processing techniques such as injection molding or extrusion permits mass production while preserving customizable properties determined by the choice of monomers, composition, surface area, and additives. This allows for the design of ocular DDSs tailored to specific patient needs and therapeutic targets. The fine-tuning leads to their versatility with predictable degradation rates and controlled drug-release characteristics. This diversity has proven effective in the delivery of various therapeutic agents for ocular conditions, including anti-inflammatory drugs, antibiotics, and antiglaucoma medications [115,116]. Their capability to encapsulate and steadily release both hydrophilic and hydrophobic drugs is an important attribute, effectively addressing common issues in ocular drug delivery associated with poor solubility.

#### 2.2.5. Biodegradable Polyurethanes

Classically synthesized from petroleum-based precursors, traditional polyurethanes (PUs) boast superior chemical stability and environmental degradation resistance, ensuring durability and product longevity. However, these same characteristics engender environmental issues as nondegradable PUs, persisting long after their utility ceases, contribute to pollution and waste-management challenges [117]. In contrast, biodegradable polyurethanes (BPUs) are crafted to disintegrate under distinct biological or environmental conditions. The degradability of BPUs chiefly hinges on the integration of biodegradable segments, frequently sourced from natural resources like vegetable oils, within the PU molecular structure [118]. The inherent versatility of BPUs allows for their properties to be precisely tuned to manifest significant elasticity and softness, typically achieved through the use of aliphatic diisocyanates. In addition, BPUs offer the flexibility for chemical modifications to introduce functional groups that can purposefully interact with drug molecules or biological entities [119]. Furthermore, their production utilizing cost-effective raw materials and moderate processing conditions leads to biomaterials that are potentially more economically efficient compared to the PLA polymers often used in biomedical applications. The biodegradability of BPUs can be engineered by incorporating hydrolysable oligomers, such as low-molecular-weight polyesters, polyethers, and poly(amino acids), as soft segments in their structure [120]. Upon hydrolysis, these tailored BPUs exhibit a notable biocompatibility, further reinforcing their utility in biomedical settings.

The deployment of BPUs within ophthalmological applications has observed an emerging growth trend in recent years. In an exploratory study by Kim et al., a polyurethane film was utilized as a sustained-release vehicle for dexamethasone during strabismus surgeries, effectively extending the postoperative adjustment period up to six weeks in rabbit eyes, thereby eliminating the need for frequent topical steroid applications [121]. In another study, Xue et al. employed biodegradable poly[(R)-3-hydroxybutyrate-(R)-3-hydroxyhexanoate]-based polyurethane thermogels as potential substitutes for injectable transparent vitreous material [122]. Further, Gisele et al. explored the synthesis of potential ocular implants designed to treat uveitis by integrating dexamethasone acetate into biodegradable polyurethanes. In vitro trials conducted during this study revealed that the biodegradable polyurethane did not release any toxic constituents [123]. These recent developments highlight the promising role that BPUs could play in future ocular treatments and interventions.

Despite their promise, the application of BPUs in the ocular field is not without challenges. Among them are the precise control of degradation rates to ensure the longevity and performance of the material within the ocular environment; the maintenance of mechanical stability throughout the degradation process to prevent premature device failure; the consideration of BPU heat sensitivity, which could limit sterilization methods and complicate manufacturing processes; and economic factors such as the potentially high costs associated with BPU synthesis, testing, and regulatory compliance, which could affect the scalability of BPU usage in ocular applications.

### 2.3. Hybrid Biodegradable Polymers

Hybrid biodegradable polymers, synergistic composites of both natural and synthetic constituents, strike an optimal equilibrium between the innate biocompatibility afforded by natural polymers and the malleability of design inherent in synthetic polymers [124]. Various exemplars have their unique merits and constraints pertinent to ocular applications. For example, PLGA/collagen hybrids merge the robustness and adaptable degradation kinetics of PLGA with the exceptional cellular compatibility conferred by collagen. This makes them suitable for ocular applications, such as scaffolds in corneal tissue engineering [125] and as biomembrane-embedded nanoparticles enabling dual-release delivery systems [126]. An additional illustration of hybrid biodegradable polymers for ocular applications includes the PLGA/chitosan composites, which harness the mucoadhesive attributes of chitosan to enhance the permeability of the incorporated drug [127]. Tahara et al. explored the delivery of therapeutics to the posterior ocular segment via a noninvasive topical application by utilizing PLGA nanoparticles surface-modified with chitosan. This modification appeared to augment the association of nanoparticles with cells, thereby enhancing delivery to the retinal segments of mice after topical administration [128]. While hybrid biodegradable polymers present compelling opportunities for ocular applications, careful consideration is needed to navigate their inherent challenges, particularly around balancing the degradation rates, mechanical properties, and potential immunogenic responses. Ongoing research is crucial to fully exploit their potential and address these limitations.

Table 1 provides a comprehensive summary of the advantages and disadvantages associated with the particular natural and synthetic biodegradable polymers delineated in the preceding discussion.

## 3. Formulation Approaches Using Biodegradable Polymers

The evolution of formulation strategies employing biodegradable polymers has spurred transformative progress in ocular drug delivery. By capitalizing on the inherent properties of these polymers, such as their biocompatibility, biodegradability, and capacity to control drug release, unique delivery vehicles have been crafted to address the specific therapeutic needs of ocular disorders.

### 3.1. Nanoparticles

Nanoparticles (NPs), colloidal entities ranging from 1 to 1000 nm, have emerged as transformative tools in ocular drug delivery due to their tunable properties, such as their charge, hydrophilicity, and hydrophobicity, along with their excellent biocompatibility, biodegradability, and stability. Constructed through the self-assembly of natural and biodegradable phospholipids in an aqueous environment, they manifest a unique bicontinuous microstructure [129]. Their suitability to a broad spectrum of biomedical applications is further enhanced by their capacity to encapsulate therapeutic compounds, thereby safeguarding them against degradation and enabling sustained release over prolonged periods. The plasticity of NPs allows for the modulation of factors such as polymer type, concentration, and crosslinking, facilitating the customization of size, encapsulation efficiency, and release dynamics to cater to ocular therapy requirements [130]. NPs can be stratified into nanospheres, where therapeutics are homogeneously dispersed in the polymer matrix, and nanocapsules, where the active compound is sequestered within the polymeric shell. Nanospheres offer superior stability and drug-loading capabilities, while nanocapsules excel in targeted delivery and preserving drug integrity [129,131].

Both natural polymers and biodegradable synthetic polymers can be employed in the formation of these drug-loaded entities. Moreover, to improve the retention of NPs on ocular surfaces, mucoadhesive nanosystems incorporating hydrophilic polymers like polyethylene glycol (PEG) and polyvinylpyrrolidone (PVP) are employed to foster interactions with mucins via hydrogen bonding and/or electrostatic forces [132]. Recent studies underscore the merits of drug-loaded NPs [133,134,135,136,137], which include superior drug retention, a reduced dosing frequency, and minimized toxicity, making them promising candidates for treating ocular surface diseases. In fact, they are poised to supplant traditional formulations as the primary treatment modality for anterior ocular diseases in the imminent future.

### 3.2. Polymeric Micelles

The utilization of polymeric micelles (PMs) as drug-delivery platforms has recently attracted substantial interest in the context of ocular disease therapy. PMs, spherical formations derived from amphiphilic polymers possessing both hydrophilic and hydrophobic fragments, exhibit remarkable attributes that optimize drug transport to targeted sites, positioning them as a viable alternative to conventional drug-delivery methods [138,139]. The polymer at the core of the PM formation is a pivotal consideration, given its significant impact on the PM stability, drug-loading potential, and drug-release traits, with the latter being influenced by the polymer’s hydrophobicity, molecular weight, and chemical composition [140]. PLGA and chitosan emerge as two frequently employed polymers in PM synthesis for drug-delivery applications. Enhancements in drug-release control and stability can be achieved by formulating PLGA with polyvinyl alcohol (PVA) [140].

The utilization of PMs in ocular drug delivery manifests several benefits. These comprise improved translocation through lipophilic cells in the corneal epithelium and endothelium; amplified interactions with the ocular surface attributable to their mucoadhesive properties; and the capacity to yield transparent aqueous solutions, simplifying the application via eye drops without hindering visual perception [141]. However, despite the promising attributes, PMs are not devoid of limitations. These encompass a low drug-loading capability, challenges in regulating the release rates, difficulties in large-scale production, and potential toxicity to ocular tissues—a concern that mandates further investigation.

### 3.3. Nanosuspensions

Nanosuspensions (NSs) are biphasic systems characterized by colloidal dispersions of nanometric drug particles, typically less than 1 µm in diameter, optimized for delivering drugs with low water solubility and absorption when administered ocularly [142]. Distinct from conventional matrix-based nanosystems, such as nanoparticles and liposomes, NSs bypass the need for carrier materials, solely consisting of pure-drug nanoparticles. These particles are stabilized with specific excipients, encompassing surfactants, viscosity modifiers, and charge modulators [143]. By transitioning a drug into NS form, its specific surface area and saturation solubility see an augmentation due to the reduced particle size. Consequently, in topical applications, a heightened bioavailability arises from an enlarged contact region, protracted drug residence duration, and elevated therapeutic concentrations within tissues, potentially necessitating reduced drug doses [144,145]. Polymers such as PCL and PLGA have been explored in NS DDSs due to their ease of preparation [146]. They contribute to a longer drug-release profile, contrasting the quicker release observed in aqueous solutions [147].

Nonetheless, NSs pose challenges. Surfactants, often used as suspending agents, might induce ocular irritation and toxicity. To mitigate such concerns, researchers are probing techniques like encapsulating NSs within appropriate gel or bioadhesive matrices or devising ophthalmic implants. These strategies aim to proffer sustained drug release while curtailing irritative or toxic risks. Furthermore, in comparison to other colloidal systems, NSs often exhibit reduced stability, limiting their storage duration. Thus, when crafting ocular drug-delivery platforms, the potential toxicity and stability of a biodegradable polymer in NSs must be meticulously assessed and refined.

### 3.4. Hydrogels

Hydrogels, created through the engineering of both natural biopolymers (such as alginate and chitosan) and synthetic polymers (including biodegradable polymers like PLA, PGA, and PLGA) via physical (ionic bonds, entanglements, hydrogen bonding, and van der Waals or hydrophobic interactions) and/or chemical (covalent bonds) crosslinking methods, are acclaimed for their significant water content and tissue-mimicking consistency [148]. In the field of recent innovations, creating polymer hydrogels are regarded as vital prospects for bioelectronic connections given their distinct fusion of electrical conduction capabilities and mechanical attributes similar to tissue [149]. This advancement even permits the utilization of 3D printing to create sophisticated interfaces for bioelectronics [150]. Another innovation includes ‘smart’ bandages with hydrogel electrodes, enabling the wireless monitoring and electrical stimulation of wounds, which has been shown to significantly enhance healing and tissue regeneration in preclinical studies [151]. These recent scholarly investigations into the preparation, performance, and fabrication techniques of hydrogels have underscored their potential as advanced delivery vehicles. The hydrophilic character, substantial hydration, and analogous mechanical properties of these three-dimensional, water-swollen matrices align closely with the characteristics of the extracellular matrix and soft tissues, which make them suitable for ocular drug delivery [152]. Given their ability to respond to stimuli like pH or temperature, they can tailor drug release contingent on ocular environmental shifts. The formulation process traditionally hinges on the physical or covalent crosslinking of hydrophilic polymers, bestowing hydrogels with both elevated water content and requisite mechanical robustness [153]. Such attributes not only protect the physicochemical state of bioactive compounds over prolonged durations, particularly in contrast to the more rigid, hydrophobic matrices prevalent in drug encapsulation, but they also forestall the degradation of peptides and proteins.

Historically, hydrogels’ versatility has been harnessed in fashioning ocular implants like punctal plugs and contact lenses, optimizing treatments for ocular surface maladies [154]. While punctal plugs have proven instrumental in ameliorating dry eye disease, the encapsulated drugs often suffer from swift depletion and a diminished bioavailability [155]. An innovative recourse entails drug encapsulation in nanocapsules or nanomicelles before integration into punctal plugs [156,157]. Alternatively, therapeutic delivery via contact lenses, typically achieved by submerging the lenses in drug solutions, has encountered issues like swift drug discharge and potential lens opacification, impairing vision [158,159,160]. A novel remedy is the ring-implanted contact lens design, encapsulating drugs within nanoparticles for release via a ring-shaped lens region, showcasing extended ocular retention and the consistent release of agents like hyaluronic acid [161]. It is anticipated that nanoparticle-infused contact lenses will pave the path for groundbreaking ocular treatments.

### 3.5. In Situ Gels

Referred to as “smart hydrogels”, in situ gels represent a unique class of hydrogels that exhibit a sol–gel transition after an in vivo application. Diverse physiological stimuli encompassing fluctuations in temperature, pH, or the ion composition within the tear or vitreous fluid can stimulate this phase transformation [162]. Upon a topical application, these less viscous solutions transform from a liquid to a gel state within the conjunctival cul-de-sac, giving rise to a bioadhesive network. This network effectively binds the medication to the ocular surface, amplifying its retention time, aiding with sustained release, and diminishing the dosing frequency, which optimizes patient compliance. In situ gels, which include both liquid and solid formulations, can be delivered via several routes. They have shown efficacy as vehicles for drug-loaded NPs, NSs, nanoemulsions, and liposomes for ocular disorder treatments [163,164].

Developing in situ gels involves straightforward steps leading to cost-effective formulations. However, limitations such as susceptibility to degradation and limited dosage incorporation need consideration [165]. Various research initiatives have aimed at preparing in situ gelling systems for sustained ocular drug delivery. Pawar et al. have, for instance, developed a thermosensitive in situ mechanism for HP-β-CD voriconazole extension by utilizing sodium alginate and Pluronic F68 [166]. In a parallel investigation, Khan et al. designed a system loaded with sparfloxacin, employing sodium alginate for gelling and methylcellulose for viscosity amplification [167]. In a comparable manner, Noreen et al. engineered a pH-responsive in situ gelation system housing moxifloxacin HCl, with Terminalia Arjuna gum and sodium alginate serving as the primary constituents [168]. Such work underscores the potential and versatility of in situ gelling systems in the realm of ocular drug delivery.

### 3.6. Biodegradable Implants

Biodegradable polymer-based implants, designed for long-term ocular drug delivery, represent an innovative class of medical therapeutics. Once positioned within ocular tissues or cavities, these systems can dispense their drug load over extended periods, thereby enhancing patient adherence and therapeutic efficacy. Although surgical implantation is generally required, the potential for controlled, protracted drug release offers an appealing alternative to recurrent, invasive intravitreal injections. The FDA currently authorizes intraocular implants as a platform for the sustained release of intravitreally administered small molecular drugs intended for the retina. These implants facilitate the regulated, sustained delivery of low-molecular-weight drugs, including both lipophilic steroids and hydrophilic substances. Two main classifications exist for these implantable devices: biodegradable and nonbiodegradable. The biodegradable polymers often employed include PLGA and PLA. Nonbiodegradable counterparts such as poly(dimethylsiloxane) (PDMS), silicone, PVA, and poly(ethylene-co-vinyl acetate) (PEVA) are also employed [169,170]. Intraocular implants fabricated from nonbiodegradable polymers demonstrate superior precision in controlling drug release and extending release durations compared to their biodegradable equivalents. However, these nonbiodegradable implants necessitate surgical procedures for both implantation and subsequent extraction, thus posing inherent surgical risks.

Numerous commercially available implants, sanctioned by the FDA for ocular disease treatment, exist in the market. The variety of these products, encompassing options like Trivaris^®^, Kenalog^®^, Iluvien^®^, Ozurdex^®^, Durysta^®^, and more, is evident in their distinct active ingredients and diverse drug-release patterns [171]. Only Ozurdex^®^ and Durysta^®^ represent biodegradable options from these available choices. Sanctioned in 2009, Ozurdex^®^, a biodegradable implant composed of PLGA, incorporates dexamethasone to address a multitude of ocular conditions such as retinal vein occlusion-induced macular edema and diabetic macular edema, with a drug-release profile spanning up to six months. Durysta^®^, greenlighted in 2020, is utilized as an intracameral injection to alleviate intraocular pressure in individuals suffering from open-angle glaucoma or ocular hypertension, lasting for a span of four to six months.

Advancements are ongoing in the development of biodegradable polymer-based implants for the sustained release of therapeutics addressing various ocular conditions. Such examples include antibiotics, antifungals, and corticosteroids, using polymers like PLGA and PLA, among others. Significantly, Brimo DDS^®^, an intravitreal implant with PLA, has cleared Phase 2 clinical trials, showcasing PLA’s gradual biodegradation in ensuring prolonged brimonidine delivery for geographic atrophy treatment [172]. Moreover, innovative photosensitive biodegradable implants like OcuLief™ and EyeLief™, developed by Re-Vana Therapeutics Ltd., are making strides [171]. As advancements in ocular disease treatments continue, the focus on biodegradable implant technologies is poised to become increasingly significant.

### 3.7. Biodegradable Nanosheets

Nanosheets, ultrathin biodegradable layered structures constituted of polyanions and polycations, offer distinct advantages like superior flexibility, nanometer-level thickness, high transparency, and impressive adhesive properties [173]. These properties, notably, can be manipulated based on thickness. The synthesis of latanoprost-loaded biodegradable nanosheets (LBNS) for ophthalmic drug delivery was pioneered by Kashiwagi et al. [173]. The production procedure harnessed differing quantities of chitosan and sodium alginate, which were layered together to construct multilayered polymeric nanosheets. These were then infused with varied amounts of latanoprost isopropyl ester. Upon application, the ensuing LBNS exhibited an ability to decrease intraocular pressure for about a week without invoking any serious adverse reactions. Wang et al. introduced an innovative dual drug-loaded nanosheet, LATINA, composed of alginate, chitosan, latanoprost, and timolol. With in vivo trials showing a consistent and slow intraocular pressure reduction, LATINA proved to be a stable, adaptable, enduring, and biocompatible biodegradable delivery mechanism [174].

### 3.8. Biodegradable Microneedles

Microneedle-based ocular drug delivery, a minimally invasive pioneering approach, can potentially transform ocular medication administration. Initially devised for transdermal delivery [175], this technology’s adaptation for ocular surfaces, such as the cornea, sclera, and suprachoroidal space, aims to evade the challenges linked to traditional ocular needle injections [176]. The primary advantage of this strategy is the significant reduction in ocular tissue damage, attributed to its minimally invasive nature. The utilization of microscaled needles (ranging 25–2000 μm in height) mitigates patient discomfort while ensuring precise drug localization [177,178]. Despite the desirable mechanical properties; ease of fabrication; and sterilization of nondegradable materials like stainless steel [118], titanium, and ceramics (aluminum oxide), their use for microneedles has a significant limitation: a lack of biocompatibility [179]. This deficiency could lead to chronic inflammation, foreign body responses, and long-term tissue damage, limiting clinical applications. As an alternative, biodegradable microneedles employ various biodegradable polymers, such as PLA, PLGA, and chitosan. The drugs are encapsulated within these microneedles, which degrade over time after application, releasing the drug in a controlled manner. The utilization of microneedles has been explored with several DDSs, including gel formulations and nanoparticle suspensions [180,181].

## 4. Biodegradable Polymer-Based Drug-Delivery Systems for Ocular Diseases

The eye’s anterior and posterior segments are susceptible to an array of vision-threatening diseases. Notably, disorders like glaucoma, anterior uveitis, and ocular surface conditions such as dry eye disease and keratoconjunctivitis predominantly impact the anterior segment. Conversely, the posterior segment is frequently compromised by conditions like age-related macular degeneration, diabetic retinopathy, and retinal vascular occlusions. The recent upsurge in attention towards biodegradable DDSs highlights their promising role in treating various ocular afflictions. They provide a targeted and prolonged release of therapeutic agents, improve drug stability and bioavailability, and offer possibilities for specific drug delivery. Consequently, this results in the overall enhancement of drug efficacy and a reduction in systemic adverse effects. These beneficial features can notably improve patient compliance and treatment outcomes, particularly in managing chronic ocular diseases that frequently require prolonged treatment. These systems are being leveraged to treat a diverse range of ocular diseases, and their application is discussed in subsequent sections in greater detail.

### 4.1. Anterior Segment Diseases

#### 4.1.1. Glaucoma

Glaucoma, a group of ocular disorders characterized by progressive optic nerve damage, stands as a leading cause of irreversible blindness globally [182]. Primarily, the condition is associated with elevated intraocular pressure (IOP), often resulting from impaired aqueous humor outflow. Despite its common occurrence, the pathogenesis of glaucoma remains complex and not fully understood. Consequently, the medical and surgical interventions currently available primarily focus on lowering IOP, which is the only modifiable risk factor to date. Pharmacological treatments for glaucoma, which encompass beta blockers, prostaglandin analogs, alpha agonists, and carbonic anhydrase inhibitors, are usually the first-line therapy. However, patient noncompliance often arises due to the inconvenience of frequent dosing, local side effects, and the asymptomatic nature of early-stage glaucoma [183]. Surgical interventions, on the other hand, can lead to complications such as hypotony, infection, and cataracts [184,185]. Laser therapy, a middle ground between pharmacological and surgical treatments, does not always provide long-term IOP control and may need to be repeated or supplemented with medication or surgery [186]. These challenges underscore the need for innovative, effective, and long-lasting glaucoma treatments.

Addressing the shortcomings of current glaucoma therapies, recent scientific focus has been geared towards the development of biodegradable polymer-based DDSs. These offer the targeted, sustained delivery of therapeutic agents to overcome the barriers presented by the eye’s unique anatomy and physiology, facilitating effective drug delivery to the anterior segment. These systems also aim to improve patient compliance by reducing the dosing frequency and minimizing systemic side effects. Numerous antiglaucoma pharmaceuticals, such as latanoprost, dorzolamide, brinzolamide, timolol maleate, brimonidine, and pilocarpine, have been the subject of research in diverse biodegradable DDSs [171]. These include polymeric NPs [187], microneedles [188], inserts [189], and in situ hydrogel systems [190,191,192,193,194,195]. For example, Franca et al. developed a chitosan/hydroxyethyl cellulose insert aimed at facilitating the sustained release of dorzolamide. Administering this ocular insert to male Wistar rats resulted in a notable decrease in the IOP for a two-week period, a change not seen in either the untreated or placebo groups. Moreover, this insert exhibited a protective effect against the death of retinal ganglion cells [189]. In a different study carried out by Pan and colleagues, they employed PLGA NPs to carry both dexamethasone and melatonin. These NPs consistently released both drugs in vitro without a burst, showed no toxicity on the R28 cells, and improved retinal penetration while significantly reducing the IOP in a rabbit eye mode [196]. In situ hydrogels, particularly those derived from gelatin, are the subject of intensive investigation for glaucoma management. A biodegradable in situ gel delivery system intended for the intracameral administration of pilocarpine was proposed by Lai et al. Gelatin-g-poly(N-isopropyl-acrylamide) was employed to produce these copolymeric carriers. The resultant carriers demonstrated a significant reduction in IOP alongside remarkable miotic effects [197]. El-Feky et al. crafted a semisynthetic chitosan–gelatin hydrogel by using oxidized sucrose, providing a sustained release of timolol for ocular hypertension control. This hydrogel, with proven mucoadhesive properties, released timolol slower than conventional eye drops, thereby extending its efficacy in male albino rabbits [193]. These formulations have shown promising results in terms of improved bioavailability and sustained drug release.

In a landmark achievement in March 2020, Durysta^®^, a PLGA-based, biodegradable, sustained-release, IOP-lowering implant, received FDA approval. Durysta^®^, a product from Allergan plc, is a rod-shaped polymer matrix housing 10 µg of bimatoprost for gradual ocular release over an extended period. The implant is designed to address nonadherence issues in glaucoma treatment, offering a prolonged, reliable, and convenient therapeutic solution [198]. Evidence for its safety and effectiveness comes from the results of two Phase III clinical studies, known as ARTEMIS 1 and 2 [199,200], and patients reported substantial implant biodegradation within a year and effective IOP control for over three years.

Durysta^®^ stands as the only approved biodegradable drug-delivery system, though many alternatives are under active exploration. For instance, ENV515 travoprost Extended Release, a rod-shaped, biodegradable intracameral implant, is fabricated by using the PRINT^®^ technique and a PLGA-inclusive polymer blend. Designed to deliver a steady supply of travoprost over 6 to 12 months, patients treated with ENV515 demonstrated similar IOP reductions to those treated with either topical travoprost 0.004% or topical timolol 0.5% [201]. Additionally, sustained IOP-reducing effects lasting 8 months after a single implantation were observed in both hypertensive and normotensive Beagle dogs in preclinical studies, affirming its safety and tolerability [202].

Ocular Therapeutix is currently investigating another biodegradable intracameral implant called the OTX-TIC. This implant comprises a soft hydrogel platform encapsulating travoprost-loaded microparticles, all maintained in a meshwork structure. A Phase 1 clinical trial evaluated the safety, effectiveness, durability, and tolerability of the OTX-TIC implant [203].

An innovative biodegradable implant called latanoprost FA SR, shaped like a rod and designed for intracameral use, is being pioneered by PolyActiva, situated in Parkville VIC, Australia. The objective is to utilize this to administer latanoprost, aiming to alleviate primary open-angle glaucoma. Currently, it is under assessment in Phase II clinical trials. The goal of these trials is to achieve a reduction in IOP by 20% within the low-dose patient group. The future trajectory in DDSs for glaucoma therapy could see the incorporation of combination therapies in a long-acting delivery device. Moreover, a ground-breaking extended-release system synchronized with a device that monitors intraocular pressure is considered advantageous.

#### 4.1.2. Anterior Uveitis

Uveitis is an inflammatory condition that affects the uveal tract, which encompasses the iris, ciliary body, and choroid. Symptoms typically include redness, pain, light sensitivity, blurred vision, and floaters. Depending on the part of the uvea affected, uveitis is classified into anterior, intermediate, posterior, and panuveitis. It can occur at any age and can be acute, recurrent, or chronic. The underlying cause can vary widely, including autoimmune disorders, infections, and injury, or it can sometimes be idiopathic. Treatment often entails the use of anti-inflammatory medications, corticosteroids, and other immunosuppressive agents [204]. Despite the availability of effective systemic and topical anti-inflammatory medications, the treatment of uveitis remains a challenge. One reason is the need for frequent dosing, which can lead to poor patient compliance and, consequently, disease recurrence. The frequent application of eye drops can also cause local side effects like cataracts and glaucoma. Moreover, the systemic administration of drugs may cause severe side effects like osteoporosis, hypertension, and gastric ulcers. This is where the potential of biodegradable DDSs comes into the picture.

Various materials are being explored for their applicability in the creation of biodegradable DDSs aimed at treating anterior uveitis. Wu et al. conducted a significant study on the use of micelles made from monomethoxy poly(ethylene glycol)-poly(ε-caprolactone), conjugated with rapamycin. When administered through an intravitreal injection, these micelles demonstrated a prolonged retention of rapamycin within the retinal pigment epithelial cells of rats, lasting for a minimum of two weeks. This prolonged release improved the treatment efficacy for autoimmune uveitis compared to the use of a rapamycin suspension alone [205].

Gonzalez-Pizarro et al. engineered an in situ gel system encapsulating fluorometholone-loaded PLGA nanoparticles. The delivery of this formulation demonstrated a noteworthy enhancement in the precorneal residency duration. This increase subsequently resulted in an amplified ocular bioavailability and deep-tissue penetration, including areas such as the aqueous humor and crystalline lens, as observed in a rabbit model [206].

In another study by Xu et al., the researchers engineered nanomicelles comprising chitosan oligosaccharide-valylvaline-stearic acid amalgamated with dexamethasone. Exhibiting prolonged drug-release characteristics, these nanomicelles additionally demonstrated enhanced adhesion to mucosal surfaces along with improved penetrative properties. Evidencing their potential efficacy, these nanomicelles manifested promising results in both rat and rabbit experimental models [207].

#### 4.1.3. Dry Eye Disease

The most recent 2017 update from the Tear Film and Ocular Surface Society’s International Dry Eye Workshop categorizes dry eye disease (DED) as a complex condition involving the ocular surface, which destabilizes the tear film and triggers eye-related symptoms [208]. The report emphasizes the significant role of tear film fluctuations, hyperosmolarity, inflammation, and damage to the ocular surface as well as neurosensory irregularities in causing DED. In some cases, it can also be associated with systemic conditions such as Sjögren’s syndrome, lupus, or rheumatoid arthritis. This condition triggers tear film instability, discomfort in the eye, vision impairments, and could potentially harm the ocular surface. Typical symptoms might manifest as a sensation of burning or stinging, impaired vision, and a feeling akin to having a foreign object or grit in the eye.

Approaches to managing DED target the replenishment or preservation of the eye’s tear volume and quality to lessen the impacts of dryness and associated discomfort. These strategies employ lubricants such as synthetic tears and sodium hyaluronate ocular drops, anti-inflammatory medications like corticosteroids and nonsteroidal anti-inflammatory drugs (NSAIDs), immune system suppressants including cyclosporine A (CsA), along with other drugs like secretagogues and autologous serum eye drops. Additionally, procedures like punctal occlusion may be applied [209,210].

Corticosteroids, recognized for their potent anti-inflammatory properties, are extensively employed to manage ocular surface inflammation. Efforts are underway to develop biodegradable corticosteroid formulations to improve bioavailability and curtail systemic side effects. Various research has successfully delivered corticosteroids like prednisolone acetate, dexamethasone sodium phosphate, fluorometholone, and triamcinolone acetonide by using delivery systems like polymeric nanoparticles, micelles, and hydrogels [134,135,136,206,211,212,213,214,215]. These systems improve the time the medication remains on the cornea and increase its availability in the eye. As an illustration, Hanafy and colleagues designed a system of self-assembled NPs loaded with prednisolone acetate by using chitosan-deoxycholate. This system managed to double the release of prednisolone after a 24 h period compared to a commercially available micronized drug-loaded gel [134]. A chitosan thermosensitive hydrogel embedded with nanostructured lipid carriers and dexamethasone was fabricated by Tan et al. [216]. The distinct feature of this concoction is its thermosensitivity, which enables it to transform into a hydrogel when interacting with the conjunctival sac upon administration as a solution into the eye. The in vitro release study findings demonstrated that this formulation enables the sustained release of dexamethasone. Beyond corticosteroids, NSAIDs also serve a significant role in managing ocular inflammation. A notable example includes the work of Sánchez-Lopez et al., who developed a dexibuprofen-loaded PLGA NP system. The NPs showed a slow two-phase release, with an initial rapid release for around 150 min followed by sustained drug release over 24 h. This demonstrated the potential for prolonged dexibuprofen delivery, which could reduce the frequency of patient dosing [133].

Topical CsA, a fungal metabolite, has gained attention due to its anti-inflammatory characteristics, which presumably stem from its capability to inhibit T-cell activation through interleukin-2. Several FDA-approved CsA-based eye drop formulations such as Restasis^®^, Ikervis^®^, Cequa^®^, and CyclASol^®^ have proven effective in alleviating symptoms and signs related to DED [155,217,218]. Significant efforts have been dedicated in recent times to leverage biodegradable DDSs to enhance the therapeutic efficacy of CsA. This innovative approach has shown encouraging outcomes. PCL NPs, augmented with penetration-boosting surfactants like benzalkonium chloride, have been employed for ocular CsA delivery [219]. Another study by Liu et al. developed surface-modified PLGA and dextran NPs with phenylboronic acid to enhance their mucoadhesive properties [220]. The findings highlighted this formulation’s enhanced safety, prolonged CsA release, and weekly dosing efficacy for inflammation mitigation and corneal healing. Eudragit RL-coated PLGA particles, known as cationic NPs, exhibited heightened corneal retention and absorption. This formulation showed substantial cellular-uptake and tear-fluid concentrations of CsA [221]. Başaran et al. developed a cationic chitosan solid-lipid NP system for CsA, which exhibited substantial precorneal retention and drug uptake. CsA was detectable in ocular fluid samples for 48 h, indicating successful penetration and prolonged release due to an increased eye residence time [222].

Research is ongoing to improve the current understanding of DED and develop novel therapeutic strategies. Although current treatment options can manage the symptoms in most patients, there are still challenges to be addressed, including identifying the exact etiology in individual patients and designing personalized therapeutic regimens.

#### 4.1.4. Keratoconjunctivitis

The widespread ocular surface disorder conjunctivitis is marked by conjunctiva inflammation and can originate from infectious sources or noninfectious elements such as allergens, toxins, or immune or neoplastic processes [223]. On the other hand, keratitis arises from corneal inflammation and can be categorized into infectious or noninfectious types based on the causative agent. Infectious keratitis can further be divided into bacterial, protozoal (e.g., Acanthamoeba), fungal, and viral forms [224].

Therapeutic strategies for keratoconjunctivitis encompass antibiotics, antivirals, antifungals, and anti-inflammatory medications tailored to address specific causative agents. However, the diminished water solubility and fleeting ocular surface residence time present notable challenges. To enhance the therapeutic efficiency, investigations into biodegradable formulations capable of protracting drug release have been conducted. Ameeduzzafar and colleagues developed chitosan-based NPs designed for the delivery of levofloxacin. These NPs displayed biocompatibility suitable for topical ocular application and exhibited an extended retention period in the ocular region in comparison to a levofloxacin solution [225]. Mudgil et al. developed a PLGA NS containing moxifloxacin. This formulation exhibited superior transcorneal permeation and exhibited a protracted antimicrobial effectiveness against Staphylococcus aureus and Pseudomonas aeruginosa, as contrasted with the commercially available eye drop Moxicip^®^ [226]. Kapanigowda et al. designed ganciclovir-loaded chitosan microspheres, which showcased a substantial amplification in peak concentration as opposed to a ganciclovir solution. The effectiveness and tolerability of the said formulation were underscored by in vivo ocular pharmacokinetic studies paired with histopathology reports [227].

Xie et al. pioneered the design of core-shell-structured HA-based microcapsules by using a one-step in situ drug-encapsulation process for ofloxacin. This unique method facilitated the construction of a resorbable hydrogel punctal plug with an enhanced and extended drug-release feature. When utilized for ofloxacin microencapsulation, it showcased improved and sustained drug release in aqueous environments [157]. Abbas et al. developed an in situ gelling solution infused with oxytetracycline-loaded gelatin-polyacrylic acid NPs [228]. This optimized solution was evaluated for its potential to counteract Pseudomonas aeruginosa through both in vitro trials and an in vivo rabbit eye conjunctivitis model. The findings suggested prolonged efficacy against keratitis and an antibacterial potency comparable to that of an established commercial product.

### 4.2. Posterior Segment Disease

#### 4.2.1. Diabetic Retinopathy

Diabetic retinopathy (DR) is a widespread microvascular issue linked to Diabetes Mellitus (DM), accounting for the majority of adult blindness between ages 20–74. Given the global increase in DM, this correlates with a growing incidence of DR. Key risk factors include disease duration, hyperglycemia levels, and hypertension. Diabetic macular edema (DME), a substantial DR subtype, is the leading cause of vision loss in DR patients and is associated with all DR severities, including nonproliferative and proliferative DR [229]. DME develops due to diabetes-induced damage to the blood–retinal barrier, causing fluid leakage into the neural retina and the subsequent thickening and cystoid edema of the macula. Antivascular endothelial growth factor (anti-VEGF) therapies and corticosteroids have a confirmed role in managing DR and DME, with recent research exploring the potential of biodegradable formulations to augment therapeutic effectiveness and bioavailability [230].

Badiee et al. designed a system that entailed the incorporation of bevacizumab-loaded chitosan NPs within a hyaluronic acid ocular implant. The obtained outcomes demonstrated that this unique formulation was capable of sustaining drug release for a duration of two months. Consequently, the utilization of this formulation presents a promising method for achieving the sustained delivery of bevacizumab [231]. Mahaling et al. undertook a study involving the administration of triamcinolone acetonide-loaded NPs, featuring a PCL core and a hydrophilic Pluronic^®^ F68 shell, in a rat model of diabetic retinopathy [232]. A marked reduction in retinal inflammation was observed, as evidenced by a decrease in the expression of NF-κB, ICAM-1, and TNFα following a 20-day treatment period. Moreover, the NP therapy resulted in attenuated glial cell hyperplasia, evidenced by the decreased expression of Glial Fibrillary Acidic Protein (GFAP). It also caused a reduction in microvascular complications, highlighted by decreased VEGF production and fewer microvascular tuft formations, observed after 40 days of therapy. Xu et al. engineered a complex of nanomicelles derived from chitosan oligosaccharide-valylvaline-stearic acid that is capable of self-assembly and the encapsulation of dexamethasone. Utilizing this approach significantly boosted access to the posterior segment of the eye via conjunctival pathways, fostered prolonged release, and heightened penetration attributes. Comparative experimental trials on male rats and albino rabbits showed comparable levels of dexamethasone to those recorded in the FDA-sanctioned NP system loaded with dexamethasone mixed with hydrogenated castor oil-40/octoxynol-40 [207]. Administering periocular injections extends the duration of drug delivery, akin to intravitreal injections, but it comes with the added benefit of a more durable injection site that minimizes the possibility of particle outflow due to tear drainage, despite the necessity for trans-scleral penetration to attain effective drug distribution. Zeng et al. designed PLGA and chitosan NPs encapsulating interleukin-12, a cytokine known for reducing MMP-9 and VEGF-A levels and inhibiting tumor angiogenesis. Despite a modest encapsulation efficiency (34.7%), the formulation demonstrated sustained drug release and a superior efficacy in inhibiting VEGF-A and MMP-9 expression in rat endothelial cells and DR mouse retinas. This formulation significantly reduced retinal damage in DR mice, as evident from the increased retinal thickness and decreased neovascularization after treatment [233].

#### 4.2.2. Age-Related Macular Degeneration

Age-related macular degeneration (AMD) is a leading cause of vision loss in industrialized nations, with severe complications like choroidal neovascularization (CNV) and geographic atrophy [234]. The favored approach for delivering anti-VEGF drugs or corticosteroids for AMD is an intravitreal injection. Yet, the permeability of therapeutic small molecules remains suboptimal. Biodegradable drug-delivery systems (DDSs) have been employed to augment the penetration of therapeutic agents across biomembranes, enhancing CNV treatment outcomes.

An innovative technique involving bilayer-dissolving microneedles loaded with ovalbumin-encapsulated PLGA NPs was proposed by Wu et al. for protein transport [235]. This method offers an ex vivo sustained protein release for more than two months and efficiently bypasses the scleral barrier, indicating its potential as a compelling strategy to treat neovascular eye conditions. In a study conducted by Varshochianand and colleagues, albumin PLGA NPs were developed to encapsulate bevacizumab. The resulting NPs offered a prolonged-release formulation of bevacizumab, which maintained a vitreous concentration exceeding 500 g/L for approximately eight weeks in a rabbit model [236]. Badiee et al., in a separate study, embedded bevacizumab into chitosan NPs that were subsequently integrated into a hyaluronic acid-derived ocular implant. While the analysis lacked in vivo testing, the laboratory-based findings revealed a prolonged two-month medication discharge period [231].

Further exploration in the field has suggested that the combined administration of dexamethasone with anti-VEGF agents, such as aflibercept and bevacizumab, in the form of polymeric NPs can exhibit a durable release profile and robust antiangiogenic effects. A case in point is the research conducted by Lui and colleagues, who crafted a unique formulation involving PLGA and polyethylenimine NPs loaded with dexamethasone, with bevacizumab adsorbed onto the surfaces. Their results indicate an enhanced antiangiogenic efficiency compared to monotherapies involving either dexamethasone or bevacizumab alone. Furthermore, this combined formulation showcased an amplified efficacy in inhibiting CNV and exhibited a strong suppressive effect on VEGF secretion [237]. Rudeen et al. innovatively designed a combination DDS that incorporates a hydrogel carrying microparticles loaded with aflibercept and NPs infused with dexamethasone. In vitro examinations of this novel formulation showed a subtle reduction in both the swelling ratio and equilibrium water content when compared to the delivery systems solely containing either aflibercept or dexamethasone. Remarkably, this combined formulation demonstrated an extended release duration, lasting up to 224 days, marking it as a potential advancement in sustained drug-delivery strategies for AMD [238].

#### 4.2.3. Retinal Vein Occlusions

Retinal vein occlusions (RVOs) represent the second most common retinal vascular disorder, second only to diabetic retinopathy. This condition includes central retinal vein occlusions (CRVOs); branch retinal vein occlusions (BRVOs); and less commonly, hemiretinal vein occlusions. Notably, BRVOs occur with a four-to-six-fold higher frequency than CRVOs, the latter of which impacts an estimated 2.5 million people globally. The incidence of RVOs is skewed towards men and those aged 65 years and above, with contributing factors encompassing age, hypertension, cardiovascular disease, DM, hyperviscosity syndromes, and glaucoma. The primary driver of progressive vision loss in RVO patients is macular edema. VEGF exacerbates this condition by promoting neovascularization and vascular permeability [239]. Additionally, a widely accepted theory underscores the role of inflammation in the progression and outcomes of vitreoretinal diseases, inclusive of RVOs [240].

In June 2009, the FDA granted approval for Ozurdex^®^, a biodegradable device manufactured by Allergan in Irvine, CA, designed to deliver 700 μg of dexamethasone to treat macular edema associated with either BRVOs or CRVOs. Data from Phase III clinical trials revealed a significant improvement in visual acuity, quantified as a gain of 15 or more letters, for a larger proportion of patients in the treatment group as compared to the sham group for up to 90 days after the injection [241]. However, this observed advantage seemed to diminish by 180 days, to the point of insignificance. Evaluations after a second round of injections at the six-month mark demonstrated a less pronounced effect by the end of the year.

A novel biodegradable dexamethasone implant, AR-1105, currently under investigation, is developed to treat macular edema resulting from CRVOs. The implant comprises a mixture of dexamethasone and a bioerodible PLGA polymer blend, fabricated by using PRINT^®^ micromolding technology. This design facilitates a more gradual release of dexamethasone at a lower total dose (340 µg) compared to existing therapies. In a multicenter Phase II trial spanning six months, two AR-1105 formulations with identical doses but varied release kinetics were evaluated for their safety and efficacy. The study yielded promising results, with both formulations exhibiting good tolerability and significant, sustained improvements in visual acuity and retinal thickness in patients with RVOs characterized by longstanding edema [242].

## 5. Conclusions

Biodegradable polymer-based DDSs offer considerable promise in the treatment of ocular diseases, heralding a new frontier in medical applications. Their potential to provide sustained and targeted drug delivery while minimizing systemic side effects renders them a highly attractive area for further exploration. However, the path towards fully realizing their potential is fraught with numerous challenges. Among these challenges are potential inflammatory responses from polymer degradation products, complexity in predicting drug-release kinetics due to variable factors, and technical issues associated with formulation and sterilization. Furthermore, the development and implementation of these systems face multifaceted regulatory obstacles that must be meticulously addressed to ensure a successful clinical translation. The regulatory standards for safety and efficacy, extensive in vivo toxicity studies, the standardization of formulation methods, and international regulatory harmonization all add complexity to the developmental process. To fully exploit the potential of these delivery systems, additional research is required to address these challenges and ensure their safety and efficacy. Collaborative approaches among researchers, clinicians, regulatory bodies, and industry stakeholders will be essential in this pursuit. As the knowledge and technology continue to advance, biodegradable polymer-based DDSs may significantly enhance the therapeutic landscape for ocular diseases.

## Figures and Tables

**Table 1:** Comparison of natural and synthetic biodegradable polymers: advantages and disadvantages.

Type of Polymer	Advantages	Disadvantages
**Natural Polymers**		
Cellulose Derivatives	Renewable, good mechanical properties	Varying solubility, can have complex purification, inconsistent degradation rates
Chitosan	Penetration enhancement, antimicrobial properties, mucoadhesive properties	Insolubility in water and alkaline media, lack of swelling properties
Hyaluronic Acid	Shear-thinning rheological characteristics, viscoelasticity, high hydration capacity, anti-inflammatory attributes, cell permeability	Expensive, susceptibility to enzymatic degradation
Gelatin	Inexpensive, enhanced solubility in aqueous systems, potent mucoadhesive characteristics, bioadhesives within ocular tissues	Thermal instability, relatively rapid degradation time
Alginate	Mucoadhesive behavior, pH-dependent gel formation ability, suitable for encapsulation	Insufficient mechanical strength and stability
**Synthetic Polymers**		
Poly(lactic acid) (PLA)	Mechanical robustness, adaptability to various processing techniques	Innate brittleness and deficient impact strength, requires specific conditions for degradation
Poly(lactic-co-glycolic acid) (PLGA)	Enhanced solubility, controlled degradation, good mechanical properties	Acidic degradation products may cause inflammation, slow degradation rate
Polycaprolactone (PCL)	Slow degradation rate, good flexibility, superior thermal stability	Limited mechanical strength, hydrophobic
Polyanhydrides (PAs)	Constant-rate drug-release kinetics, low toxicity profile of their degradation byproducts, surface erosion degradation pattern	Susceptibility to hydrolytic degradation, complexity in synthesis, potential challenges with mechanical properties
Biodegradable Polyurethanes	Flexibility for chemical modifications, economically efficient	Potential toxicity issues, heat sensitivity, degradation products and rates can vary significantly

## Data Availability

Not applicable.
